# Paralytic Shellfish Toxins Occurrence in Non-Traditional Invertebrate Vectors from North Atlantic Waters (Azores, Madeira, and Morocco)

**DOI:** 10.3390/toxins10090362

**Published:** 2018-09-06

**Authors:** Marisa Silva, Verónica Rey, Aldo Barreiro, Manfred Kaufmann, Ana Isabel Neto, Meryem Hassouani, Brahim Sabour, Ana Botana, Luis M. Botana, Vitor Vasconcelos

**Affiliations:** 1 Department of Biology, Science Faculty, University of Porto, Rua do Campo Alegre, 4619-007 Porto, Portugal; aldobarreiro@gmail.com (A.B.); vmvascon@fc.up.pt (V.V.); 2 Interdisciplinary Center of Marine and Environmental Research-CIMAR/CIIMAR, University of Porto, Novo Edificio do Terminal de Cruzeiros do Porto de Leixões, Avenida General Norton de Matos, 4450-208 S/N Matosinhos, Portugal; mkaufmann@ciimar.up.pt; 3 Department of Analytical Chemistry, Science Faculty, University of Santiago de Compostela, 27002 Lugo, Spain; veronica.rey@rai.usc.es (V.R.); anamaria.botana@usc.es (A.B.); 4 Life Sciences Faculty, Madeira University, Marine Biology Station, 9000-107 Funchal, Madeira Island, Portugal; 5 Center of Interdisciplinary Marine and Environmental Research of Madeira-CIIMAR-Madeira, Edificio Madeira Tecnopolo, Caminho da Penteada, 9020-105 Funchal, Madeira, Portugal; 6 cE3c/GBA—Centre for Ecology, Evolution and Environmental Changes/Azorean Biodiversity Group and Department of Biology, Faculty of Sciences and Technology, University of Azores, 9501-801 Ponta Delgada, São Miguel, Azores, Portugal; ana.im.neto@uac.pt; 7 Phycology Research Unit-Biotechnology, Ecosystems Ecology and Valorization Laboratory, Science Faculty, University of Chouaib Doukkali, El Jadida BP20, Morocco; hassouani@hotmail.com (M.H.); sabour.b@ucd.ac.ma (B.S.); 8 Department of Pharmacology, Veterinary Faculty, University of Santiago de Compostela, 27002 Lugo, Spain; luis.botana@usc.es

**Keywords:** paralytic shellfish toxins, new vectors, post-column oxidation liquid chromatography, Madeira island, São Miguel island, Morocco

## Abstract

Paralytic shellfish toxins (PSTs) are potent alkaloids of microalgal and cyanobacterial origin, with worldwide distribution. Over the last 20 years, the number of poisoning incidents has declined as a result of the implementation of legislation and monitoring programs based on bivalves. In the summer of 2012 and 2013, we collected a total of 98 samples from 23 different species belonging to benthic and subtidal organisms, such as echinoderms, crustaceans, bivalves, and gastropods. The sampling locations were Madeira, São Miguel Island (Azores archipelago), and the northwestern coast of Morocco. The samples were analyzed using post-column oxidation liquid chromatography with a fluorescence detection method. Our main goal was to detect new vectors for these biotoxins. After reporting a total of 59 positive results for PSTs with 14 new vectors identified, we verified that some of the amounts exceeded the limit value established in the EU. These results suggest that routine monitoring of saxitoxin and its analogs should be extended to more potential vectors other than bivalves, including other edible organisms, for a better protection of public health.

## 1. Introduction

Paralytic shellfish toxins (PSTs) are a type of phycotoxins which represent a serious threat to public health. Episodes of poisoning in humans are caused not only by bivalve consumption but also by other sorts of seafood. They are produced by several common genera of microalgae and cyanobacteria with worldwide distribution. They are a group of alkaloids including saxitoxin (STX) and its analogs. To date, more than 50 saxitoxin analogues have been identified and are divided into five groups [1]: (i) carbamate toxins (saxitoxin, neosaxitoxin (NEO), and gonyautoxins (GTX1-4)); (ii) decarbamoyl toxins (decarbamoyl-saxitoxin (dcSTX), decarbamoyl-neosaxitoxin (dcNEO) and decarbamoyl-gonyautoxins (dcGTX1-4)); (iii) N-sulfocarbamoyl-toxins (gonyautoxins 5-6 (GTX5-6) and C1-4) cited from higher to lower toxicity; (iv) 13-deoxydecarbamoyl toxins (13-deoxydecarbamoyl-saxitoxin (doSTX) and 13-deoxydecarbamoy-gonyautoxins (doGTX2-3)); (v) GC toxin (GC1–6), with unknown toxic properties [2,3].

Marine dinoflagellates of the genera *Alexandrium*, *Gymnodinium*, and *Pyrodinium* are considered the main producers, being the majority of cases of paralytic shellfish poisoning (PSP) associated with blooming events of these species [4,5]. Although PSTs production has also been found in freshwater and brackish cyanobacteria [6], these organisms are rarely reported as the original cause of human intoxications.

PSTs are spread through the food web by the ingestion of their producers, i.e., the above-mentioned dinoflagellate genera, and bioaccumulation in seafood. Contaminated seafood is found mainly in filter-feeding mollusks like bivalves, in particular mussels and clams [4]. Although bivalves are considered the traditional vectors and bioindicators for PSTs, reports of non-traditional vectors are being increasingly recognized. These include marine gastropods (carnivorous and grazers), crustaceans, echinoderms, tunicates, and ascidians, which can accumulate toxic levels of PSTs [7,8,9,10,11]. Certain fish such as sardines [12], salmon [13,14], herring [15], and mackerel [16,17] were also reported to accumulate PSTs, though only sub-toxic levels have been detected so far [8].

Even though a large number of PSP cases have been reported all over the world [4,5,18], the successful implementation of monitoring programs for the presence of PSTs in both marine microalgae and shellfish and an action level or regulatory limit defined as 800 µg STX equivalents (eq)diHCl/kg tissue [19] have helped to minimize public health risks in many countries.

For the last 70 years, the mousse bioassay [20] has been used worldwide as an official method in monitoring programs. While this method is considered reliable for regulatory purposes, it is being replaced by chemical methods that provide better results in terms of reproducibility and toxin profile detection. There are two methods for PSTs analysis (based on the Association Official Analytical Chemists (AOAC)-validated High Pressure Liquid Chromatography with Fluorescence Detection (HPLC-FLD)) that have been successfully validated in a collaborative study, according to the harmonized protocol of ISO/IUPAC/AOAC [21] for detection and quantification of PSTs in mussels, clams, scallops, and oysters. These two methods consist in a pre-column oxidation method [22] and a post-column oxidation method (PCOX) [23]. Although the pre-column oxidation method (method of Lawrence) may also be used as an alternative method to the mouse bioassay [24], the PCOX has been proposed as a better routine option [25].

European reference methods for the determination of PSTs were developed and optimized for the analysis of bivalve mollusks. Nevertheless, the increase in the production and capture of certain species for which monitoring programs have not been properly designed is being gradually recognized because of commercial demands in the fishing industry. However, for non-bivalve vectors, no specific monitoring plans have been established to date [26].

The production or capture of echinoderms and tunicates in the EU is small compared to those of fish and other seafood [13]. The commercialization of gastropods and crustaceans has been growing in the last few years [14]. According to FAO reports from recent years [27], crustacean fisheries exceeded the production of bivalve mollusks in the EU, and marine gastropod fisheries have also increased considerably, duplicating in the last two decades.

Therefore, bearing in mind the growing consumers’ interest in these species, it is of special importance to evaluate the exposure to marine biotoxins through the ingestion of these new vectors as well as the adjustment of the existing reference methods for this new scenario. In this work, samples of different invertebrate and fish species from the North Atlantic waters were analyzed using the PCOX method in order to detect new vectors for these biotoxins and evaluate their potential as a threat to public health.

## 2. Results and Discussion

A total of 98 samples were analyzed using the PCOX method [10] with slight modifications [28] to determine and quantify PSTs. The samples were collected from three different sites: Madeira Island, São Miguel Island (Azores archipelago), and the Moroccan Atlantic coast (the sampling sites are described in detail in the Material and Methods section). Several edible (with commercial interest) and non-edible species were selected to search for potential new vectors and the prevalence of the screened biotoxins in the food web: gastropods (*Stramonita haemastoma, Phorcus lineatus*, *Cerithium vulgatum*, *Gibbula umbilicalis*, *Aplysia depilans*, *Charonia lampas*, *Onchidella celtica*, *Patella gomesii*, *Patella aspera, Umbraculum umbraculum, Patella ordinaria*), crustaceans (*Pollicipes pollicipes*), bivalves (*Mytilus* spp.), starfish (*Ophidiaster ophidianus*, *Marthasterias glacialis*, *Echinaster sepositus*), sea-cucumber (*Holothuria* (*Platyperona*) *sanctori*), sea-urchins (*Paracentrotus lividus*, *Arbacia lixula*, *Sphaerechinus granularis*, *Diadema africanum*), and fish (*Sphoeroides marmuratus*).

Since the PCOX method is not validated for these different matrices, optimizations were needed and made for echinoderms and gastropods species, thus adding an additional step prior to the HPLC-FLD analyses and enhancing the reliability of the results [29].

### 2.1. Madeira Island (Madeira Archipelago)

From a total of 22 samples collected during the summer of 2012, 15 were positive for PSTs, with 7 above the maximum legislated value [30,31] (Table 1).

Regarding toxin uptake, the highest values were detected in echinoderms, more specifically, in the red velvet starfish *O. ophidianus* (4625.4 µg STX.diHCleq/Kg), followed by gastropods *C. lampas* (1423.4 µg STX.diHCleq/Kg SM), *P. ordinaria* (1123.3 µg STX.diHCleq/Kg SM), and *S. haemostoma* (964.5 µg STX.diHCleq/Kg SM).

Concerning monitoring, bivalves are the selected key sentinel species for which all analytical methods have been validated [22,23,26]. Being the Madeira archipelago located in oligotrophic waters where mussels are quite rare, echinoderms and gastropods showed a good potential as bio-indicators for toxin monitoring, as suggested in previous works [11,32,33].

### 2.2. São Miguel Island (Azores Archipelago)

From a total of 38 samples from June 2013, 22 were positive for PSTs, with 7 above the maximum legislated value [30,31] (Table 2).

Regarding toxin uptake (Table 2), similarly to Madeira, we detected seven values above the legal limit in starfish (*O. ophidianus* and *M. glacialis*), followed by mollusks *S. haemostoma* and *P. gomesii* (939.4 and 902.3 µg STX.diHCleq/Kg SM, respectively). The maximum uptake value detected was in the yellow spiny starfish *M. glacialis* from Cruzeiro, with 7744.3 µg STX.diHCleq/Kg.

### 2.3. Moroccan Coast

The northwestern Moroccan coast was surveyed during July 2013, supplying a total of 38 samples, with 28 of them (74%) positive for saxitoxin and its analogs (Table 3).

All positive values were found in mollusks and echinoderms. Thirteen were above the European [30,31] maximum legal limit. It is important to notice that the highest concentration value detected was in a limpet, with a total amount of 3622.5 µg STX.diHCleq/Kg SM, despite the presence of mussels in this region.

### 2.4. Statistical Analysis

The results of the generalized linear model for PST content as a function of genus and region (*Patella* and *Paracentrotus*) and region (Madeira and Morocco) did not find significant differences for region, despite the southwards increase detected in the whole data set. This could be explained by the low number of samples and organisms included and by the fact that these two sampling regions are closer to each other, relative to Azores. On the other hand, a significant difference was detected in toxin concentrations between genera, with higher concentrations in *Patella* than in the sea urchin (F_1,16_ = 10.4, *p* < 0.01), which seems to be a general pattern for sea urchins and limpets in all the three regions (see Table 1, Table 2 and Table 3).

### 2.5. General Discussion

**Figure 1:**
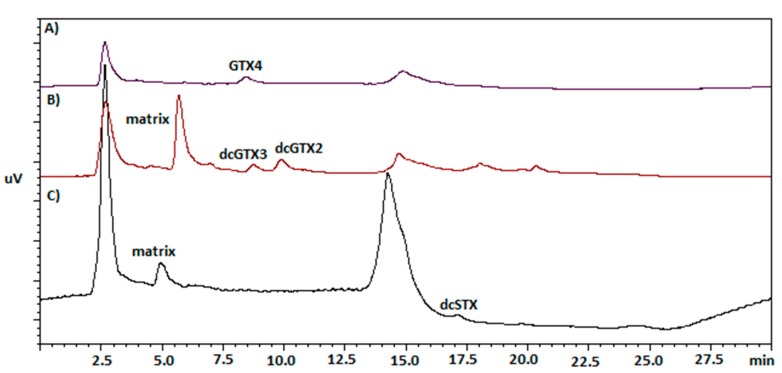
Comparison of chromatographic separations of gonyautoxins and saxitoxins: (A) Sample 485, *Mytillus* spp. (B) Sample 443, *Stramonita haemostoma*. (C) Sample 484, *Pollicipes pollicipes*. Obtained by the post-column oxidation (PCOX) method.

One of the primary aims of this work was to screen new vectors for PSTs in order to evaluate public health threats related to seafood consumption. From a total of 66% of positive results, we report 14 new vectors for these hydrophilic phycotoxins, belonging to three different phyla: mollusks (*P. aspera*, *S. haemostoma*, *U. umbraculum*, *P. gomesii*, *P. ordinaria*, *C. vulgatum*, *O. celtica*), echinoderms (*O. ophidianus*, *A. lixula*, *E. sepositus*, *D. africanum*, *S. granularis*, *H. (Platyperona) sanctori*), and crustaceans (*P. pollicipes*). In Figure 1 are displayed some examples of the toxin elution in different matrices, showing that the toxin retention time was dependent on the analyzed matrix used in the PCOX method.

**Figure 2:**
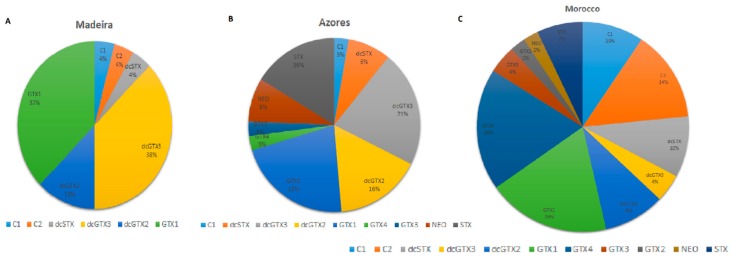
Average toxin frequency in Madeira island (**A**), Azores (**B**), and Morocco (**C**).

We highlight the latitudinal pattern of PSTs uptake, since the percentage of positive results follows a north-south gradient: Azores (58%) < Madeira (68%) < Morocco (74%). Though many of the causes of dinoflagellate bloom formation are still to unravel, the water temperature and eutrophication play a pivotal role, which is consistent with our results. Water temperature in the Atlantic Ocean rises, in general, following a north-south gradient towards the Equator, and human anthropic pressures are higher in Morocco than in the Portuguese islands screened in this survey [34,35,36,37,38,39]. We also defined a qualitative pattern of toxin profiles, as shown in Figure 2.

Among the different saxitoxin derivatives, dcGTX3 and GTX1 were more frequent in Madeira, dcGTX2, dcGTX3, GTX1, and STX in São Miguel island from Azores, and C1, C2, GTX1, and GTX4 in the Moroccan Coast. As explained in the introduction, the Toxicity Equivalency Factors (TEFs) of PSTs are: carbamate group > decarbamoyl group > N-sulfocarbamoyl group. According to this classification, the average PSTs toxin profiles from the Portuguese islands were more toxic than those from the Moroccan coast [40]. One of the reasons that could contribute to this trend is the vector species analyzed from each sampling site, since it is known that PSTs are subject to conversions in the digestive tracts of their vectors [41,42]. The Madeira and Azores profiles could be more similar to each other than to the Moroccan coast profile because of the higher percentage of echinoderms and gastropods sampled. Also, bivalves are quite rare in the Portuguese islands.

All the crustacean samples were positive for PSTs, although the detected values were below the legal limit. These group of organisms are not considered in the European Legislation for marine toxins monitoring [43], although one of the species analyzed, *P. pollicipes* (see chromatogram in Figure 1), is of high commercial value. Regarding starfish, we reported 14 positive samples for PSTs, and nine of them exceeded the legal limit. In prior studies, PSTs were also detected in these matrices, though in smaller amounts and always below the legal European limit [11,44,45,46]. It is important to note that, to the best of our knowledge, we reported here the presence of PSTs in the holothuridae group for the first time [26]. Regarding PSTs detection in species with commercial value other than mussels, we found quantifiable amounts of these alkaloids in *P. gomesii*, *P. aspera*, *P. pollicipes*, *P. lividus*, and *C. lampas* (values displayed in Table 1, Table 2 and Table 3). It is important to point out sample 336 purchased in a local market in Madeira island, which exhibited an amount of PSTs above the legal limit (1123.3 µg STX.diHCl eq/Kg SM). Limpets are a much appreciated delicacy, mainly in the Madeira archipelago. Previous studies showed the need to revise and update the legislation regarding these organisms, i.e., monitoring practices should be mandatory, and analytic practices should be optimized for these vectors to avoid human poisoning incidents [26]. Our work shows real risks to human consumers, as our analyses were performed in purchased samples. In addition, since bivalves are absent from the Portuguese islands, echinoderms and gastropods would be a suitable alternative as toxin bioindicators in these geographical areas [11,32,33].

The European reference methods for PSTs determination, based on liquid chromatography with fluorescence detection [22,23], were developed and optimized for bivalves, though echinoderms and gastropods are also contemplated in the monitoring programs. In this work, we demonstrated that, with small amendments, it is possible to optimize with reliability the available methods for these matrices (Figure 1). Also, the application to crustaceans was straightforward. To summarize, the PCOX method [23] has proved to be a good technique for analyzing PSTs in non-traditional vectors.

## 3. Conclusions

In this study, we used the PCOX technique to screen non-traditional vectors for paralytic shellfish toxins in three sampling areas: Madeira island, São Miguel island (Azores archipelago), and the northwestern Moroccan coast. In a total of 98 samples, 60% were positives, with the PST content of 27 samples above the maximum legislated EU value. We report for the first time 14 vectors for PSTs *(P. aspera, S. haemostoma, U. umbraculum, P. ordinaria, P. gomesii, C. vulgatum, O. celtica, O. ophidianus, A. lixula, E. sepositus, D. africanum, S. granularis, H. (Platyperona) sanctori*, and *P. pollicipes*).

PST positivity was found both in edible and in non-edible species. Non-edible species are also important because they could transfer toxins to other compartments of the food web and, eventually, to edible species. Monitoring non-edible species, like mussels in the Portuguese archipelagos, could also be important, since the legislated bioindicator is rare or absent. In this sense, echinoderms and gastropods could be a good alternative for toxin monitoring in these locations. Positive hits in high-value commercial species (e.g., *Patella aspera*, *Pollicipes pollicipes*, *Charonia lampas*) highlight the need to reinforce monitoring programs with the inclusion of these species.

## 4. Materials and Methods

### 4.1. Sampling Sites and Selected Species

**Figure 3:**
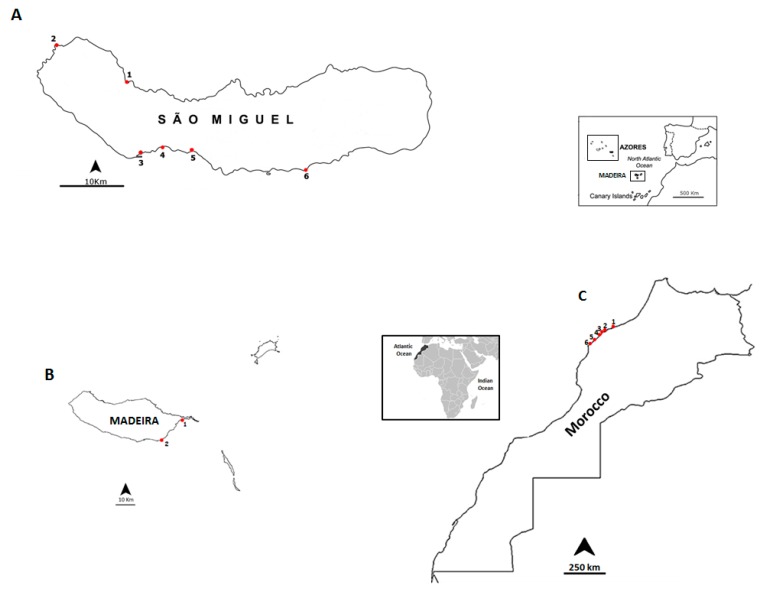
Location of the sampling sites: (**A**) São Miguel island coast, Azores archipelago: 1, Cruzeiro; 2, Mosteiros; 3, Étar; 4, São Roque; 5, Lagoa; 6, Caloura. (**B**) Madeira island coast: 1, Reis Magos and 2, Caniçal. **(C)** Northwestern Moroccan coast: 1, Casablanca Corniche; 2, El Jadida Haras; 3, El Jadida Sâada; 4, Sidi Bouzid; 5, Mrizika; 6, Oualidia.

The Portuguese islands of Madeira (Madeira archipelago), São Miguel (Azores archipelago), and the northwestern coast of Morocco where surveyed for non-traditional vector species for PSTs (Figure 3). Benthic organisms were harvested from the intertidal areas during low tide and by scuba diving expeditions. Madeira island was surveyed in September 2012. São Miguel island (Azores) and the Moroccan coast were sampled in June and July of 2013, respectively. The sampling sites are shown in Table 4. The samples of *P. ordinaria* and *P. aspera* were purchased in local markets in Madeira, caught in the northern coast of the island (32°51′17.02″ N; 17°01′54.02” W). The organisms were transported to the laboratory in refrigerated containers. The samples that were not immediately processed were carefully stored at −20 °C.

### 4.2. PSTs Extraction and Analysis Method

#### 4.2.1. Chemicals and Solutions

Analytical reagent-grade hydrochloric acid 37%, nitric acid 65%, ortho-phosphoric acid 85%, glacial acetic acid, periodic acid, sodium hydroxide, sodium dihydrogen phosphate; HPLC-grade methanol and acetonitrile were from Panreac Quimica S.A. (Barcelona, Spain). Trichloroacetic acid, tetrabutyl ammonium phosphate, ammonium hydroxide 28–30%, 2-mercaptoethanol (2-ME), and heptane sulfonate were purchased from Sigma-Aldrich (Madrid, Spain).

The certified standards provided by Cifga S.A. (Lugo, Spain) were: saxitoxin (STX), neosaxitoxin (NEO), decarbamoyl-saxitoxin (dcSTX), gonyautoxins 1 and 4 (GTX1,4), gonyautoxin 5 (GTX5), gonyautoxins 2 and 3 (GTX2,3), decarbamoyl-gonyautoxins 2 and 3 (dcGTX2,3), and N-sulfocarbamoyl-gonyautoxins 2 and 3 (C1 and C2).

#### 4.2.2. Apparatus

The LC system used, composed of a binary LC-10A pump system, a degasser DGU-14A, a fluorescence detector RF-10AXL, an autoinjector SIL-20AC with a refrigerated rack, a column oven CTO-20AC, and a system controller CBM-20A, was from Shimadzu (Izasa, Barcelona, Spain). The Shimadzu LC solution software was used to control the system.

The post-column reaction system used was formed by a knitted reaction coil with 1 mL of total volume (5 m × 0.50 mm i.d., Supelco, Madrid, Spain) immersed in a water bath at 80 °C, and two post-column pumps, LC-20AD and LC-6A and was from Shimadzu (Izasa, Barcelona, Spain).

#### 4.2.3. Extraction Protocol

The samples were extracted following the PCOX method for the extraction of PSTs with proper amendments, adjusted to the type of sample tested [1]. Briefly, the animals were dissected and homogenized with a blender (A320R1, 700 W, Moulinex, Lisbon, Portugal) in pooled groups in order to obtain 1 g of extractable tissue, with the exception of *C. lampas*, *M. glacialis*, *A. depilans*, *H.* spp., *O. ophidianus*, *S. granularis*, *U. umbraculum*, *D. antilarium*, *S. marmuratus*, which were treated individually, since each animal had enough extractable biomass. A 1 g sample was homogenized with 1 mL of 0.1 M of HCl using a vortex mixer. The pH was adjusted, between 2 and 4, with 5 M of HCl or 5 M of NaOH, while stirring the mixture. The sample was heated in a boiling water bath for 5 min and cooled to room temperature. The pH was re-adjusted, and the samples was remixed and centrifuged again at 3000× *g* for 10 min (Centric150, Tehtnica, Spain). The supernatant was decanted into a 15 mL falcon, then 500 µL were collected and deproteinated with 25 µL of 30% (*v*/*v*) TCA. The contents were mixed in a vortex mixer and centrifuged at 16,000× *g* for 5 min (Biocen 22, Ortoalresa, Madrid, Spain). An amount of 40 µL of 1 M NaOH was added, and the sample was mixed and centrifuged at 16,000× *g* for 5 min. The supernatant was filtered through a 0.2 µm nylon syringe filter (Whatman, Cambridge, UK) into a vial for analysis. Aliquots (5 µL for C toxins and 10 µL for the GTX and STX toxins group) were injected into the system [47].

#### 4.2.4. HPLC Analysis

The post-column oxidation and fluorescence detection method [48] was used with the modifications reported by Rey et al. (2015) [28].

C toxins were separated using a 25 cm × 4.6 mm i.d., 5 µm BetaBasis C8 column (Thermo, Fisher Scientific, Madrid, Spain), with a column oven at 20 °C. Solvent A was composed of 2 mM of tetrabutyl ammonium phosphate aqueous solution. Solvent B was composed by 2 mM of tetrabutyl ammonium phosphate in 4% of acetonitrile. Both solvents had their pH adjusted to 5.8 with 1% of NH_4_OH. The gradient started with 0% of B during the first 8 min, which increased to 100% up to minute 15, was maintained at this concentration for one minute, then decreased to 0% in 3 min and remained at 0% for 5 min before the next injection.

STX and GTX toxins were separated using a 15 cm × 4.6 mm i.d., 3.5 µm Zorbax Bonus-RP column (Agilent Technologies, Madrid, Spain), with a column oven at 30 °C. Solvent A was composed of 8.25 mM of heptane sulfonate and 5.5 mM of H_3_PO_4_ aqueous solution. Solvent B was 8.25 mM heptane sulfonate and 16.5 mM H_3_PO_4_ in 11.5% of acetonitrile. Both solvents had their pH adjusted to 7.1 with 28–30% of NH_4_OH. The gradient used was 0% B over 8.4 min, 100% B at 8.5 min for 10 min, and 0% B at 18.5 min for 9 min before the next injection.

#### 4.2.5. Post-Column Derivatization

The flow rate was 0.8 mL/min for both separations; after leaving the column, the eluate was mixed in a T connection with the oxidant: 100 mM H_3_PO_4_, 5 mM H_5_IO aqueous solution, adjusted to pH 7.8 with 5 M NaOH; the oxidant flow rate was 0.5 mL/min. The resulting mix was heated while passing through a knitted teflon coil (5 m × 0.50 mm i.d.) immersed in the water bath at 80 °C. Thereafter the resulting mixture was acidified in other T connection with 0.1 M of nitric acid at a flow rate of 0.3 mL/min, to reach a pH outflow between 5 and 7 [49]. The resulting derivatives were screened using a fluorescence detector at 330 and 395 nm excitation and emission wavelengths, respectively. Before analysis of both groups of toxins, the LC system was equilibrated with 100% of solvent A for at least 20 min at a 0.8 mL/min flow rate, with an oxidant flow rate of 0.5 mL/min and an acid flow rate of 0.3 mL/min. All solutions and post-column reagents were filtered through a 0.2 µm nylon filter membrane before use.

#### 4.2.6. Toxin Identification and Quantification

The C toxins standards were diluted in deionized water (DIW) and adjusted to pH 5 with 10% of acetic acid. The STX and GTX standards were diluted in 0.003 M HCl. Calibration solutions were prepared by diluting the standard stocks in toxin-free shellfish tissue extracts for STX and GTX toxins and in DIW (pH = 5) for C toxins. GTX and STX toxins and C toxins stock and working solutions were stored at 4 °C and below −20 °C, respectively [50]. For GTX and STX toxins, working standard solutions were prepared using the matrix extracts corresponding to the type of sample, i.e., each type of organism analyzed in this work (starfish, gastropods, limpets, bivalves, crustaceans, sea-urchins, fish, and sea-cucumbers) to assist in the identification of toxins present in the samples. The identification of PSP toxins was done by comparing the retention times of the samples with those of the working solutions. Each PSP toxin was quantitatively determined by direct comparison of the peak areas in the samples with those of the standards, using the corresponding calibration curves. All results were corrected for the method dilution. The total toxicity of a sample was calculated by using TEFs from Oshima (1995) and expressed as µg STX∙diHCl eq/kg [40].

Several samples of gastropods and starfish (codes 351, 353, 354, 412, 424, 428, 440, 443, 454, 470, 474, 475, 477, 483) underwent a reduction reaction using 2-mercaptoethanol to confirm the presence of GTX4 in the samples’ extracts. The protocol described by Silva & Rey was used [29]: the samples’ extracts were mixed with 1 M 2-ME in 0.1 M phosphate buffer, pH 7.4. An aliquot of this mixture was analyzed by HPLC-FLD before and after the reductive reaction for direct comparisons. The mixture was heated at 100 °C for 30 min in a water bath and analyzed by HPLC-FLD. The concentration of GTX4 was calculated after this reductive reaction from the concentration of NEO formed, using the equations described by Silva & Rey [29].

### 4.3. Statistical Analyses

The patterns of PSTs content, across invertebrate species and regions, were analyzed for the genera *Patella* and *Paracentrotus* and the regions Morocco and Madeira. This choice was made because only these two genera were found with enough replicates in the two regions (but not in Azores). ‘PST content’ (µg STX.diHCleq/Kg Shellfish Meat) was used as a dependent variable, and ‘genus’ and ‘region’ as fixed factors. Because the PST content was not normally distributed, we implemented a generalized linear model with Gamma distribution for the error term. This model was executed with the *glm* function from the *R* package *stats*. Outliers were removed with the Bonferroni test, implemented in the *outlierTest* function from the *R* package *car*. An analysis of deviance was performed to test for the main effects of the model terms, executed with the *Anova* function from the *R* package *car*.

## Figures and Tables

**Table 1:** Quantified paralytic shellfish toxins (PSTs) samples in Madeira island, Madeira Archipelago.

Sampling Data	Sampling Site	Sample	Species	Code	µg STX.diHCleq/Kg SM
8 August 2012	Northern coast of Madeira	Limpet	*Patella ordinaria*	336	1123.3
		Limpet	*Patella aspera*	337	122.4
16 September 2012	Reis Magos	Sea urchin	*Paracentrotus lividus*	339#1	<LOQ
		Starfish	*Ophidiaster ophidianus*	341#1	2071
		Starfish	*O. ophidianus*	341#2	2224.1
		Starfish	*O. ophidianus*	341#3	4625.4
		Limpet	*P. aspera*	344	866.4
		Sea urchin	*Arbacia lixula*	345	<LOQ
		Sea snail	*Stramonita haemastoma*	346	964.5
18 September 2012	Caniçal	Limpet	*P. aspera*	350	12.2
		Limpet	*Umbraculum umbraculum*	351	536.8
		Starfish	*Echinaster sepositus*	353	668
		Sea snail	*Charonia lampas*	354	1423.4
		Sea urchin	*Diadema africanum*	355#1	276.3
		Sea urchin	*D. africanum*	355#2	227.9

Values in bold are above the legal limit; STX: saxitoxin; eq: equivalents; SM: Shellfish Meat; LOQ: Limit of quantification.

**Table 2:** Information on Azores samples.

Sampling Data	Sampling Site	Sample	Species	Code	µg STX.diHCleq/Kg SM
7 June 2013	Lagoa	Sea urchin	*Sphaerechinus granularis*	409#3	43.4
		Sea urchin	*S. granularis*	409#4	42.5
		Starfish	*O. ophidianus*	412	1689.6
		Sea snail	*S. haemastoma*	413	939.4
		Limpet	*Patella gomesii*	415	1192.4
	Mosteiros	Limpet	*P. gomesii*	420	902.3
8 June 2013	Etar	Sea urchin	*A. lixula*	421	<LOQ
	Ilhéu S. Roque	Sea urchin	*A. lixula*	423	111.7
		Starfish	*O. ophidianus*	424	2588.4
		Sea urchin	*S. granularis*	425#1	<LOQ
		Sea urchin	*S. granularis*	425#2	<LOQ
		Sea urchin	*S. granularis*	425#3	<LOQ
		Starfish	*Marthasterias glacialis*	426#2	3.8
9 June 2013	Cruzeiro	Starfish	*M. glacialis*	428	7744.3
		Sea snail	*S. haemastoma*	431	678.3
	Caloura	Sea urchin	*A. lixula*	432	<LOQ
		Starfish	*M. glacialis*	433#1	47.5
		Starfish	*M. glacialis*	433#2	24.9
		Sea snail	*S. haemastoma*	434	544.7
		Starfish	*O. ophidianus*	435	920.3
10 June 2013	Caloura	Starfish	*O. ophidianus*	440	245
10 June 2013	Caloura	Sea snail	*S. haemastoma*	443	128.6

Values in bold are above the legal limit.

**Table 3:** Moroccan samples information.

Sampling Data	Sampling Site	Sample	Species	Code	µg STX.diHCleq/Kg SM
22 July 2013	Casablanca Corniche	Bivalve	*Mytilus* sp.	447	1376.9
		Sea snail	*Phorcus lineatus*	448	929.4
23 July 2013	Sidi Bouzid	Sea snail	*P. lineatus*	449	1404.5
		Limpet	*Patella* sp.	450	1090.5
		Sea slug	*Aplysia depilans*	451	<LOQ
		Bivalve	*Mytilus* sp.	453	2266.4
		Sea snail	*Cerithium vulgatum*	454	158.8
	El Jadida Sâada	Sea snail	*C. vulgatum*	455	2556
		Sea cucumber	*Holothuria (Platyperona) sanctori*	458#1	2.3
		Limpet	*Patella* sp.	459	8.6
		Starfish	*M. glacialis*	463	1852.4
24 July 2013	El Jadida Haras	Barnacle	*Pollicipes pollicipes*	464	17.7
	Mrizika	Bivalve	*Mytilus* sp.	465	1140.4
		Barnacle	*P. pollicipes*	466	17.6
		Limpet	*Patella* sp.	467	3622.5
		Bivalve	*Mytilus* sp.	468	1080.9
		Sea snail	*Gibbula umbilicalis*	469	1.6
		Sea snail	*P. lineatus*	470	1043.9
		Starfish	*M. glacialis*	473	1325.4
		Sea slug	*Onchidella celtica*	474	38.8
	Oualidia	Sea snail	*C. lampas*	475	0.02
		Sea slug	*A. depilans*	476	0.6
		Sea snail	*S. haemastoma*	477	384
		Sea snail	*P. lineatus*	482	85.7
		Sea snail	*G. umbilicalis*	483	12.1
		Barnacle	*P. pollicipes*	484	17.4
		Bivalve	*Mytilus* sp.	485	2708.9

Values in bold are above the legal limit.

**Table 4:** Sampling sites and respective geographical coordinates, surveyed during September 2012 and June and July 2013.

Date	Location	Sampling Site	Geographic Coordinates
September 2012	Madeira Island	Reis Magos	32°39′16.21″ N; 16°49′05.29″ W
		Caniçal	32°44′20.08″ N; 16°44′17.55″ W
June 2013	São Miguel Island	Cruzeiro	37° 50′31.19″ N; 25° 41′33.61″ W
		Étar	37°44′19.31″ N; 25°39′38.84″ W
		São Roque	37°45′15.35″ N; 25°38′31.60″ W
		Mosteiros	37°53′25.57″ N; 25°49′14.72″ W
		Lagoa	37°44′42.38″ N; 25°19′.47″ W
		Caloura	37°42′49.34″ N; 25°29′54.54″ W
July 2013	Morocco Coast	Casablanca corniche	33°36′01.2″ N; 7°39′57.5″ W
		El Jadida Haras	33°14′42.0″ N; 8°28′37.5″ W
		El Jadida Sâada	33°14′42.4″ N; 8°32′26.9″ W
		Sidi Bouzid	33°13′57.1″ N; 8°33′20.9″ W
		Mrizika	32°57′21.8″ N; 8°46′53.2″ W
		Oualidia	32°43′55.8″ N; 9°02′57.6″ W

## References

[1] Wiese M., D’Agostino P., Mihali T., Moffitt M., Neilan B.A. (2010). Neurotoxic alkaloids: Saxitoxin and its analogues. Mar. Drugs.

[2] Negri A.P., Stirling D.J., Quilliam M., Blackburn S., Bolch C., Burton I., Eaglesham G., Thomas K., Walter J., Willis R. (2003). Three novel hydroxybenzoate saxitoxin analogues isolated from the dinoflagellate Gymnodinium catenatum. Chem. Res. Toxicol..

[3] Vale P. (2008). Complex profiles of hydrophobic paralytic shellfish poisoning compounds in Gymnodinium catenatum identified by liquid chromatography with fluorescence detection and mass spectrometry. J. Chromatogr. A.

[4] Shumway S.E. (1990). A review of the effects of algal blooms on shellfish and aquaculture. J. World Aquacul. Soc..

[5] Shumway S.E. (1995). Phycotoxin-related shellfish poisoning: Bivalve mollusks are not the only vectors. Rev. Fish. Sci..

[6] Kellmann R., Ploux O., Neilan B.A., Ramawat K., Merillon J. (2013). Neurotoxic alkalids from cyanobacteria. Natural Products-Phytochemistry, Botany and Metabolism of Alkaloids, Phenolics and Terpenes.

[7] Bricelj M.V., Shumway S.E. (1998). Paralytic shellfish toxins in bivalve moluscs: Ocurrence, transfer kinetics and biotransformation. Rev. Fish. Sci..

[8] Deeds J.R., Landsberg J.H., Etheridge S.M., Pitcher G.C., Longan S.W. (2008). Non-traditional vectors for paralytic shellfish poisoning. Mar. Drugs.

[9] García C., Pérez F., Contreras C., Figueroa D., Barriga A., López-Rivera A., Araneda O.F., Contreras H.R. (2015). Saxitoxins and okadaic acid group: Accumulation and distribution in invertebrate marine vectors from Southern Chile. Food Addit. Contam. Part. A.

[10] Zamorano R., MarãN M., Cabrera F., Contreras C., Barriga A., Lagos N., García C. (2013). Determination of the variability of both hydrophilic and lipophilic toxins in endemic wild bivalves and carnivorous gastropods from the Southerm part of Chile. Food Addit. Contam. Part. A.

[11] Silva M., Barreiro A., Rodriguez P., Otero P., Azevedo J., Alfonso A., Botana L.M., Vasconcelos V. (2013). New Invertebrate Vectors for PST, Spirolides and Okadaic Acid in the North Atlantic. Mar. Drugs.

[12] Costa P.R., Botelho M.J., Lefebvre K.A. (2010). Characterization of paralytic shellfish toxins in seawater and sardines (*Sardina pilchardus*) during blooms of *Gymnodinium catenatum*. Hydrobiologia.

[13] Cembella A.D., Quilliam M.A., Lewis N.I., Bauder A.G., Dell’Aversano C., Thomasa K., Jellet J., Cusack R.R. (2002). The toxigenic marine dinoflagellate *Alexandrium tamarense* as the probable cause of mortality of caged salmon in Nova Scotia. Harmful Algae.

[14] Sephton D.H., Haya K., Martin J.L., LeGresley M.M., Page F.H. (2007). Paralytic shellfish toxins in zooplankton, mussels, lobsters and caged Atlantic salmon, *Salmo salar*, during a bloom of *Alexandrium fundyense* off Gran Manan Island, in the Bay of Fundy. Harmful Algae.

[15] White A.W., Ragelis E.P. (1984). Paralytic shellfish toxins and finfish. Seafood Toxins.

[16] Haya K., Martin J.L., Waiwood B.A., Burridge L.E., Hungerford J.M., Zitko V., Graneli E., Sundstrom B., Edler L., Anderson D.M. (1990). Identification of paralytic shellfish toxins in mackerel from southwest Bay of Fundy, Canada. Toxic Marine Phytoplankton.

[17] Castonguay M., Levasseur M., Beaulieu J.L., Gregoire F., Michaud S., Bonneau E., Bates S.S. (1997). Accumulation of PSP toxins in Atlantic mackerel: Seasonal and ontogenetic variations. J. Fish. Biol..

[18] Shumway S.E., Barter J., Sherman-Caswell S. (1990). Auditing the impact of toxic algal blooms on oysters. Environ. Audit..

[19] EU Council (2004). Regulation (EC) No 853/2004 of the European Parliament and of the Council of 29 April 2004 laying down specific hygiene rules for food of animal origin. Off. J. Eur. Union.

[20] Horwitz W., AOAC (2000). Paralytic shellfish poison. Method 958.08. Official Methods of Analysis os AOAC International.

[21] Horwitz W. (1995). Protocol for the design, conduct and interpretation of method-performance studies. Pure Appl. Chem..

[22] Horwitz W., AOAC (2005). Official Method 2005.06. Paralytic Shellfish Poisoning Toxins in Shellfish. Prechomatographic Oxidation and Liquid Chromatography with Fluorescence Detection. First Action 2005. Official Methods of Analysis of AOAC International.

[23] AOAC (2011). Official Method 2011.02. Determination of Paralytic Shellfish Poisoning Toxins in mussels, clams, oysters and scallops. Post-column oxidation method (PCOX). First action 2011. Official Methods of Analysis.

[24] EU Council (2005). Regulation (EC) No 2074/2005 of 5 December 2005, laying down implementing measures for certain products unde Regulation (EC) No 853/2004 of the European Parliament and of the Council and for the organisation of official controls under Regulation (EC) No 854/2004 of the European Parliament and of the Council and Regulation (EC) No 882/2004 of the European Parliament and of the Council, derogating from the Regulation (EC) No 852/2004 of the European Parliament and of the Council and amending Regulations (EC) No 853/2004 and (EC) No 854/2004. Off. J. Eur. Union.

[25] Rey V., Botana A.M., Botana L.M. (2017). Quantification of PSP toxins in toxic shellfish matrices using post-column oxidation liquid chromatography and pre-column oxidation liquid chromatography methods suggest post-column oxidation liquid chromatography as a good monitoring method of choice. Toxicon.

[26] Costa P.R., Costa S.T., Braga A.C., Rodrigues S.M., Vale P. (2017). Relevance and challenges in monitoring marine biotoxins in non-bivalve vectors. Food Control..

[27] FAO (2015). Fishery and aquaculture statistics. Global Capture Production 1950–2013 (FishstatJ).

[28] Rey V., Alfonso A., Botana L.M., Botana A.M. (2015). Influence of Different Shellfish Matrices on the Separation of PSP Toxins Using a Postcolumn Oxidation Liquid Chromatography Method. Toxins.

[29] Silva M., Rey V., Botana A., Vasconcelos V., Botana L.M. (2016). Determination of Gonyautoxin-4 in Echinoderms and Gastropod Matrices by Conversion to Neosaxitoxin Using 2-Mercaptoethanol and Post-Column Oxidation Liquid Chromatography with Fluorescence Detection. Toxins.

[30] Alexander J., Auðunsson G.A., Benford D., Cockburn A., Cravedi J.P., Dogliotti E., Di Domenico A., Fernández-Cruz M.L., Fink-Gremmels J., Fürst P. (2009). Scientific Opinion of the Panel on Contaminants in the Food Chain on a request from the European Commission on Marine Biotoxins in Shellfish-Saxitoxin Group. Efsa J..

[31] Regulation (EC) No 854/2004 of the European Parliament and of the Council of 29 April 2004 Laying Down Specific Rules for the Organisation of Official Controls on Products of Animal Origin Intended for Human Consumption. http://eur-lex.europa.eu/legal-content/EN/TXT/?uri=CELEX:32007R1246.

[32] Silva M., Azevedo J., Rodriguez P., Alfonso A., Botana L.M., Vasconcelos V. (2012). New Gastropod Vectors and Tetrodotoxin Potential Expansion in Temperate Waters of the Atlantic Ocean. Mar. Drugs.

[33] Silva M., Rodriguez I., Barreiro A., Kaufmann M., Neto A.I., Hassouani M., Sabour B., Alfonso A., Botana L.M., Vasconcelos V. (2015). First Report of Ciguatoxins in Two Starfish Species: *Ophidiaster ophidianus* and *Marthasterias glacialis*. Toxins.

[34] Hallegraeff G.M. (1993). Algal blooms are not a simple toxic broth. Search.

[35] Hallegraeff G.M. (2010). Ocean climate change, phytoplankton community responses, and harmful algal blooms: A formidable predictive challenge. J. Phycol..

[36] Anderson D.M., Glibert P.M., Burkholder J.M. (2002). Harmful Algal blooms and eutrophication: Nutrient sources, composition, and consequences. Estuaries.

[37] Burkholder J.M., Glibert P.M., Skelton H.M. (2008). Mixotrophy, a major mode of nutrition for harmful algal species in eutrophic waters. Harmful Algae.

[38] Glibert P., Seitzinger S., Heil C.A., Burkholder J.M., Parrow M.W., Codispoti L.A., Kelly V. (2005). The role of eutrophication in coastal proliferation of harmful algal blooms: New perspectives and new approaches. Oceanography.

[39] Heisler J.P., Gilbert J., Burkholder J., Anderson D., Cochlan W., Dennison W., Dortch Q., Gobler C.J., Heil C., Humphries E. (2008). Eutrophication and harmful algal blooms: Scientific consensus. Harmful Algae.

[40] Oshima Y. (1995). Postcolumn derivatization liquid chromatographic method for paralytic shellfish toxins. J. AOAC Int..

[41] Asakawa M., Takagi M., Iida A., Oishi K. (1987). Studies on the conversion of paralytic shellfish poison (Psp) components by biochemical reducing agents. Eisei Kagaku.

[42] Oshima Y., Lassus P., Arzul G., Erard E., Gentien P., Marcaillou C. (1995). Chemical and Enzymatic Transformation of Paralytic Shellfish Toxins in Marine Organisms. Harmful Marine Algal Blooms.

[43] Regulation (EC) No. 853/2004 of the European Parliament and of the Council of 29 April 2004 Laying Down Specific Hygiene Rules for Food of Animal Origin. http://eur-lex.europa.eu.

[44] Asakawa M., Nishimura F., Miyazaki K., Noguchi T. (1997). Occurrence of Paralytic Shellfish Poison in the starfish *Asteria amurensis* in Kure Bay, Hiroshima prefecture, Japan. Toxicon.

[45] Ito K., Asakawa M., Sida Y., Miyazaki K. (2003). Occurrence of paralytic shellfish poison (PSP) in the starfish *Asterina pectinifera* collected from the Kure Bay, Hiroshima Prefecture, Japan. Toxicon.

[46] Lin S.J., Tsai Y.H., Lin H.P., Hwang D.F. (1998). Paralytic toxins in Taiwanese starfish *Astropecten scoparious*. Toxicon.

[47] Van de Riet J.M., Gibbs R.S., Chou F.W., Muggah P.M., Rourke W.A., Burns G., Thomas K., Quilliam M.A. (2009). Liquid chromatographic post-column oxidation method for analysis of paralytic shellfish toxins in mussels, clams, scallops, and oysters: Single-laboratory validation. J. AOAC Int..

[48] Anon A. (2011). Official Method 2011.02 Determination of Paralytic Shellfish Poisoning Toxins in Mussels, Clams, Oysters and Scallops; Post-column Oxidation Method (PCOX).

[49] Vale C., Alfonso A., Vieytes M.R., Romarís X.M., Arévalo F., Botana A.M., Botana L.M. (2008). In vitro and in vivo evaluation of paralytic shellfish poisoning toxin potency and the influence of the pH of extraction. Anal. Chem..

[50] Rourke W.A., Murphy C.J., Pitcher G., van de Riet J.M., Burns B.G., Thomas K.M., Quilliam M.A. (2008). Rapid postcolumn methodology for determination of paralytic shellfish toxins in shellfish tissue. J. AOAC Int..

